# Health Tract, No. 112

**Published:** 1862-12

**Authors:** 


### HEALTH TRACT, No. 112.

### SOLDIER ITEMS.

SWALLOWING POISON.—Stir in a glass of water a heaping teaspoonful each of salt and kitchen mustard, and drink it instantly—this will empty the stomach in a minute. To antagonize any poison that may be left, swallow the whites of two or three eggs; then drink a cup or two of very strong coffee, or as much sweet milk or cream, if impossible to get coffee.

POISONED VINES.—Apply a paste made of gunpowder, or sulphur, with milk; renew night and morning, until cured. Live on gruel, soups, rice, and other mild food, having the bowels to act twice a day.

SIGNS OF DEATH.—Bury no man unless his head is off, or the abdomen begins to turn green or dark, the only sure signs, but always sure, of actual death. If there is haste, cut off a toe or finger, which would wake up the slightest spark of life left.

TO STOP BLEEDING.—Four or five drops of Perchlorideof Iron will check completely the flow of blood from all except the largest arteries; half a teaspoonful will arrest even their bleeding. Each non-commissioned officer should have two ounces of this in a flat tin bottle, wound around with a little cotton batting, on a bit of which the liquid could be dropped for application.

OBEDIENCE is not servility, it is a high duty; it is not cowardly, but proudly honorable in a soldier. If your officer speaks sharply, it is neither to insult nor to browbeat; it is to wake up attention, instant and implicit.

FOR every wounded soldier taken to the hospital in the Crimean war, twelve were taken on account of disease; disease which could be avoided in more than half the cases by such care as the soldier can take of himself, as directed in these pages. Of the 15,000 lives lost in the Mexican war, only 1548 were from battle. The United States Sanative Commission report that 104 soldiers became sick to each 1000 in the present war.

SHIRTS.—A distinguished British Army Surgeon says: More than one half of all army diseases in warm countries are owing to the exposure of the abdomen to changes of temperature. Shirts should reach the thigh.

INNER CLOTHING.—Every garment which touches a soldier's skin should be woolen in all seasons, most important in the warmest weather. It is impossible to over-estimate the value of this one item to the health of an army.

LIMESTONE-WATER.—One teaspoonful of vinegar, in a pint of such water, will antagonize all its ill effects on the bowels of those unaccustomed to it.

DIRTY WATER.—As much powdered alum as will rest on a dime, stirred in a pail of water, will clarify it in five minutes.

SAVING LIFE.—In the first seven months of the Crimean campaign, the soldiers died at the rate of 60 out of a 100 per annum, while for the last five months of the war not so many soldiers died of disease as at home, owing to a more systematic and rigid attention to five things: 1st. Selecting healthful camps; 2d. Enforcing strict cleanliness; 3d. Avoiding *unnecessary* exposure; 4th. Proper preparation of healthful food; 5th. Judicious nursing.

A TRUE SOLDIER is considered one of the highest types of a man. But that officer merits not the name or the title he bears, who does not make the comfort and health of his men a subject of unceasing thought, and of the most indefatigable effort.

CAMP-GROUNDS.—An elevation is a hundred-fold better than a flat or a hollow; open ground better than among trees; better for health, safer from surprise, and stronger for attack and defense, even if it is calculated to stay but a few hours. Let the tent face the south, the top screened with brushwood, and if practicable with a floor of boards three inches above the ground, and a ditch around the tent six or eight inches deep.

DRINKING WATER improperly has killed thousands of soldiers. If possible, avoid drinking any thing on a march. If you must drink, the colder the water the less will it satisfy thirst. ☞ Half a glass of water drank in sips, swallowing each sip, with a few seconds interval, will more effectually satisfy thirst, and that without any danger, than a quart taken in the usual manner at one draught. It is greatly safest, *while marching*, to rinse the mouth only, but do that to the utmost extent desired, spirting out the water as soon as it becomes warm. Chewing even a stick or pebble moderates thirst.

MITTENS, for cold weather, should have a thumb and one finger, the other three fingers together, so as to use the trigger handily.

BOWEL AFFECTIONS are said to be cured, if at all curable, by drinking from one half to four half pints of a tea made of the inner bark of the sweet-gum tree, boiled until of the taste and color of strong coffee, with or without sugar, cold or hot. The tree abounds southward.

SABBATH-DAY PARADES.—Immorality and irreligion are among the great evils of war. Knowing this, every Christian should be most dili-gent, not only in prayer for the soldiers, and in furnishing them with religious privileges in the camp, but in cherishing a strong and enlightened public religious sentiment. Public sentiment is a powerful stimulant to moral principle, as well as to patriotic feeling. It hence becomes the whole Christian community to frown upon Sabbath-day parades and displays.

